# Molecular details of secretory phospholipase A_2_ from flax (*Linum usitatissimum* L.) provide insight into its structure and function

**DOI:** 10.1038/s41598-017-10969-9

**Published:** 2017-09-11

**Authors:** Payal Gupta, Prasanta K. Dash

**Affiliations:** 1 ICAR-National Research Centre on Plant Biotechnology, Pusa Campus, New Delhi, 110012 India; 2 Department of Biotechnology, Kurukshetra University, Thanesar, 136119 India

## Abstract

Secretory phospholipase A_2_ (sPLA_2_) are low molecular weight proteins (12–18 kDa) involved in a suite of plant cellular processes imparting growth and development. With myriad roles in physiological and biochemical processes in plants, detailed analysis of sPLA_2_ in flax/linseed is meagre. The present work, first in flax, embodies cloning, expression, purification and molecular characterisation of two distinct sPLA_2_s (I and II) from flax. PLA_2_ activity of the cloned sPLA_2_s were biochemically assayed authenticating them as *bona fide* phospholipase A_2_. Physiochemical properties of both the sPLA_2_s revealed they are thermostable proteins requiring di-valent cations for optimum activity.While, structural analysis of both the proteins revealed deviations in the amino acid sequence at C- & N-terminal regions; hydropathic study revealed LusPLA_2_I as a hydrophobic protein and LusPLA_2_II as a hydrophilic protein. Structural analysis of flax sPLA_2_s revealed that secondary structure of both the proteins are dominated by α-helix followed by random coils. Modular superimposition of LusPLA_2_ isoforms with rice sPLA_2_ confirmed monomeric structural preservation among plant phospholipase A_2_ and provided insight into structure of folded flax sPLA_2_s.

## Introduction

Utility of flax (*Linum usitatissimum*) to mankind as a valued fibre dates back to more than 8000 years in history^1^. However, as of today, it has become a multipurpose crop that serves as a source of high quality fiber “linen”- a widely used raw material for textile industry. Additionally, the seed oil is an excellent source of omega-3-fatty acid ALA (α-linolenic acid, C:18)^2^. The flax seed is also a repository of active lignan compound “Secoisolariciresinol diglycoside (SDG)”^3^ that exhibit health benefits. The oil from flax seed is an excellent industrial solvent being used in paints, and varnishes. Innumerable use of flax increases its value in commercial market making it a high value cash crop. On the other hand, phospholipases have been known to have role in industrial applications amongst which secretory phospholipase A_2_ (sPLA_2_) from animals and microbes have been reported to be used in food industry in emulsification and degumming^4^. However, no industrial application of sPLA_2_ from plant source is reported as yet^5^.

Phospholipase A_2_ belongs to a group of hydrolases (EC: 3.1.1.4) that stereo-specifically catalyses the hydrolysis of second acyl ester bond of phospholipids generating free fatty acids (FFAs) and lysophospholipids (LPLs)^6^. Based on the structure, function and evolution, PLA_2_s are classified into 15 groups (I-XV) belonging to five major types. Amongst them calcium-dependent and independent cytosolic phospholipase A_2_, platelet-activating factor acetyl hydrolases, lysosomal phospholipase A_2_ and secretory phospholipase A_2_ are important^7^. Plant secretory PLA_2_s belong to group XI, which is further sub-divided into XIA and XIB^6^. In plants, sPLA_2_s are ubiquitous and are extensively studied enzymes known to be involved in a suite of signal transduction pathways^8, 9^. They play myriad roles in biological and metabolic processes leading to growth^10^, development^11, 12^ including plant defence^13^ and imparting tolerance against abiotic stress^14, 15^.

Secretory phospholipase A_2_ are low molecular weight proteins (12–18 KDa) characterised by a N-terminal signal peptide, with 12 conserved cysteine residues that form six intra-molecular disulphide bridges^16^. Functional domain of sPLA_2_s constitutes a signature phospholipase A_2_ (PA2c) domain, which is highly conserved from animals to plants^16^. This domain is characterized by presence of a conserved Ca^2+^ binding loop (YGKYCGxxxxGC) and a catalytically active motif (DACCxxHDxC) containing conserved His/Asp dyad in the active site. These conserved motifs are necessary for the catalytic activity of sPLA_2_^16^. His residue at the catalytic site motif is required for nucleophilic attack at the sn-2 acyl bond of phospholipids^17^. These enzymes are heat stable and require micro to milli molar concentration of Ca^2+^ for optimum activity^16^.

Although sPLA_2_s have been identified and purified from a number of plants, detailed functional information about these enzymes in flax is lacking. Among plants, the structure of arabidopsis (*Arabidopsis thaliana*) sPLA_2_ isoform α and soybean (*Glycine max*) sPLA_2_ isoform II has been elucidated by homology modelling. Currently, tertiary structure of sPLA_2_ isoform II from rice (OssPLA_2_II) is available and is the only testimony (PDB entry 2WG7A)^18^ of sPLA_2_s from plants. However, three-dimensional structures of flax sPLA_2_s are hitherto not available in any database.

Flax is an economically important field crop. Nearly one-fifth of its transcriptome is unique^19^ and that motivated us to characterize the novel secretory PLA_2_ enzymes of flax and perform detailed structural and functional analysis of LusPLA_2_s (flax sPLA_2_I & II). Both sPLA_2_s in flax were characterised at molecular level by cloning and expressing purified LusPLA_2_I and LusPLA_2_II in native form. The PLA_2_ activity of both flax sPLA_2_s was bio-assayed using LOX/PLA_2_ based reaction to authenticate them as *bona fide* phospholipases. In the present study, we cloned, biochemically characterized the flax sPLA_2_ proteins. Further, we investigated the three-dimensional structure of both the phospholipases of flax based on homology modelling and elucidated the topology of calcium binding loop and catalytic motif site. Our study is the first comprehensive biochemical and molecular analysis providing insight into folded secretory phospholipase A_2_ of flax.

## Results

### Discerning sequence, function, domain and cellular localization of flax sPLA_2_s

Domains are the functional units of a protein and are known to be evolutionarily conserved^20^. Our exploration of plant specific secretory phospholipase A_2_ led to identification of two sPLA_2_ in flax. A single PA2c domain, the signature domain for sPLA_2_ (with accession no. cd04706 and PSSM ID: 153095), was identified in LusPLA_2_I as well as in LusPLA_2_II proteins that belonged to superfamily cd05417domain (see Supplementary Table S1).

**Figure 1:**
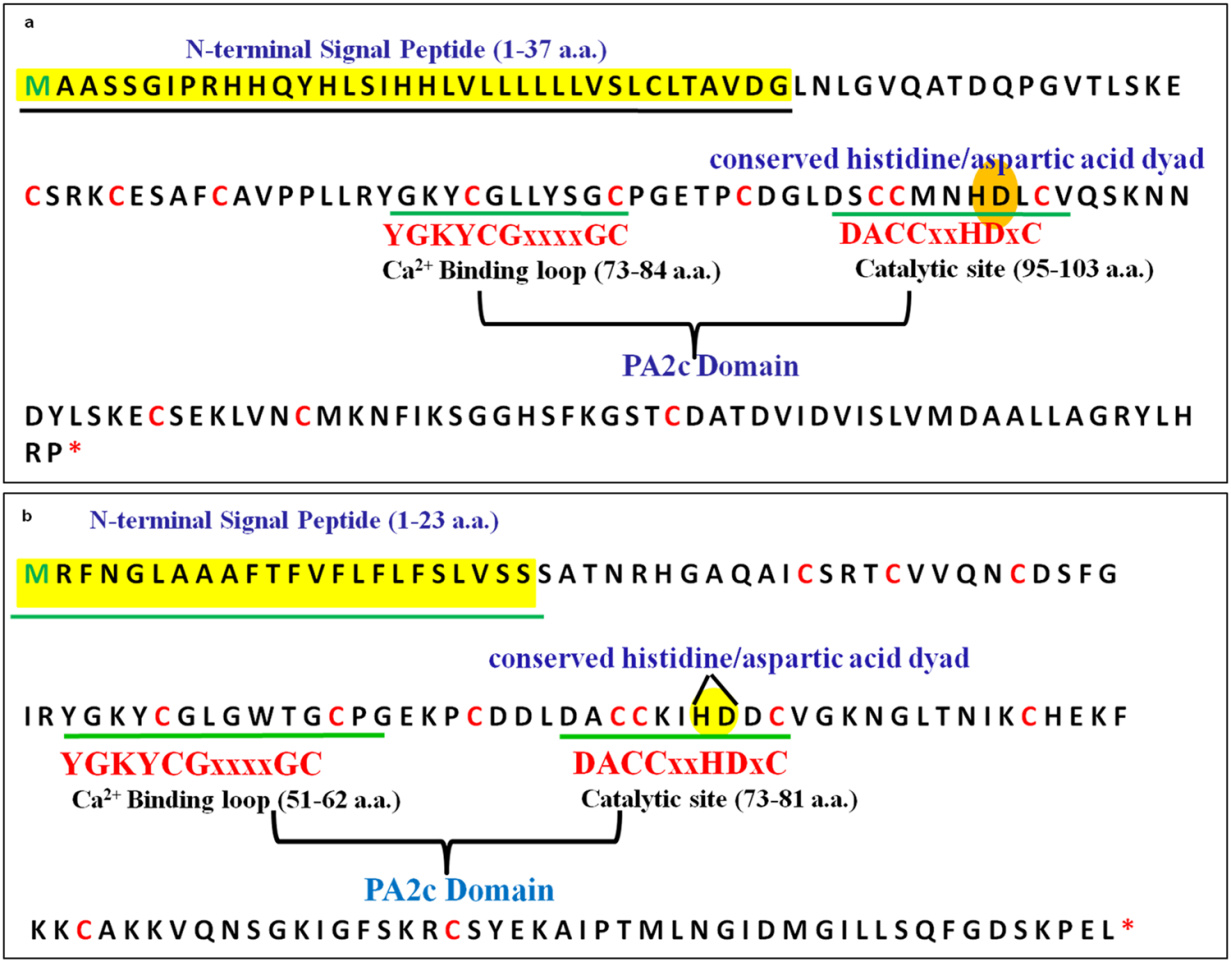
Characteristic structural features of secretory phospholipase A_2_ identified in flax sPLA_2_I and II. (**a**) Characteristic features of secretory phospholipase A_2_ identified in LusPLA_2_I. (**b**) Characteristic features of secretory phospholipase A_2_ identified in LusPLA2II. N-terminal signal peptide is highlighted in yellow, conserved calcium binding loop and catalytic site are underlined in green and represented by sequence in red, conserved His/Asp dyad is represented in oval shape, and twelve conserved cysteine residues are shown in red colour.

LusPLA_2_s contained two motifs in the PA2c signature domain, viz. Ca^2+^ binding motif and catalytic active-site motif characterized by the presence of conserved His/Asp dyad (Fig. 1a,b). These motifs are highly conserved in plant sPLA_2_s^16^. We observed, Ala residue present in the highly conserved catalytic domain (LDACCxxHDxCV) of sPLA_2_s is replaced by a Ser residue (LDSCCMNHDLCV) in flax sPLA_2_ isoform II. Twelve conserved Cys residues were identified at positions Cys56, Cys60, Cys65, Cys77, Cys84, Cys90, Cys96, Cys97, Cys103, Cys116, Cys123, Cys140 in LusPLA_2_I and Cys35, Cys39, Cys44, Cys55, Cys62, Cys68, Cys74, Cys75, Cys81, Cys92, Cys99, Cys115 in LusPLA_2_II (Fig. 1a,b). Other conserved amino acids involved in Ca^2+^ binding and catalysis of the substrate were also present in both sPLA_2_s in flax^6^.

To ascertain (% similarity/dissimilarity) all the plant sPLA_2_ sequences from flax (LusPLA_2_), rice (OssPLA_2_), arabidopsis (AtsPLA_2_), soybean (GmsPLA_2_), wheat (TdsPLA_2_, *Triticum durum*) and snake venom (*Naja kaouthia*) were aligned using Mafft-win and ESPRIPT. All the sPLA_2_s from plant origin showed a close evolutionary relationship (see Supplementary Fig. S1). The alignment of amino acid sequence among the known plant sPLA_2_s revealed that LusPLA_2_I belonged to group XIB with maximum similarity to soybean (isoform I and II) and arabidopsis (sPLA_2_ α) while LusPLA_2_II belonged to XIA with maximum relatedness to rice isoform I along with arabidopsis sPLA_2_ β, γ, δ.

We explored occurrence of potential signal peptide in the LusPLA_2_s by using the SignalP and target organelle by TargetP tools. SignalP results suggested that LusPLA_2_I possessed a 37 amino acid (Met1- Gly37) signal peptide at N-terminal end and is cleaved to generate a 129 amino acids long holoenzyme after cleavage. Similarly, LusPLA_2_II contained a 23 amino acid (Met1- Ser23) signal peptide at N-terminal end and generates the holoenzyme of 121 amino acids (see Supplementary Fig. S1). TargetP software (http://www.cbs.dtu.dk/services/TargetP/output.php) predicted LusPLA_2_I to be secreted into the extracellular space with a reliability class of 4 while iPSORT predicted mitochondria as the target organelle. This prediction in extracellular space and mitochondria can be ascribed to re-localization of sPLA_2_s upon interaction with other genes or external stimuli. Similarly, TargetP (with a probability of 0.9) predicted LusPLA_2_II to be secreted into the extracellular space with a reliability class 2.

Gene ontological classification of flax sPLA_2_s suggested that these proteins are involved in biological and metabolic processes, including anabolism and catabolism associated with growth and development (Table 1). Apart from growth and development, LusPLA_2_I and LusPLA_2_II are involved in biological processes such as response to external stimuli and internal stimuli, wounding and pathogen attack, abiotic and biotic stress response. They are also found to be involved in molecular functions like protein/receptor binding, metal ion binding and catalytic activity of substrate (Table 1).

**Table 1:** Gene ontology classification of secretory sPLA_2_ (LusPLA_2_I and LusPLA_2_II) from flax.

Biological process	Molecular function	Cellular component
Lipid catabolic process	phospholipase A_2_ activity	Extracellular region
Growth and development	calcium ion binding	Endoplasmic reticulum
Wounding and pathogen attack	Hydrolase activity	Intracellular organelle
Abiotic and biotic stress	Metal ion binding	Membrane bound
Phospholipid metabolic process	Catalytic activity	Organelle
Primary metabolic process	Protein binding	Cytoplasm
Cellular process	Receptor binding	Endomembrane system
Single-organism metabolic process	Lipase activity	Cell part
Phosphate containing		Cell surface
Anatomical structure		Golgi apparatus
morphogenesis		Intracellular part
Organogenesis		
Response to hormone		
Response to external stimuli		
Biological regulation		
Response to endogenous stimuli		

### Cloning, expression and purification of *LusPLA*_*2*_*s*

**Figure 2:**
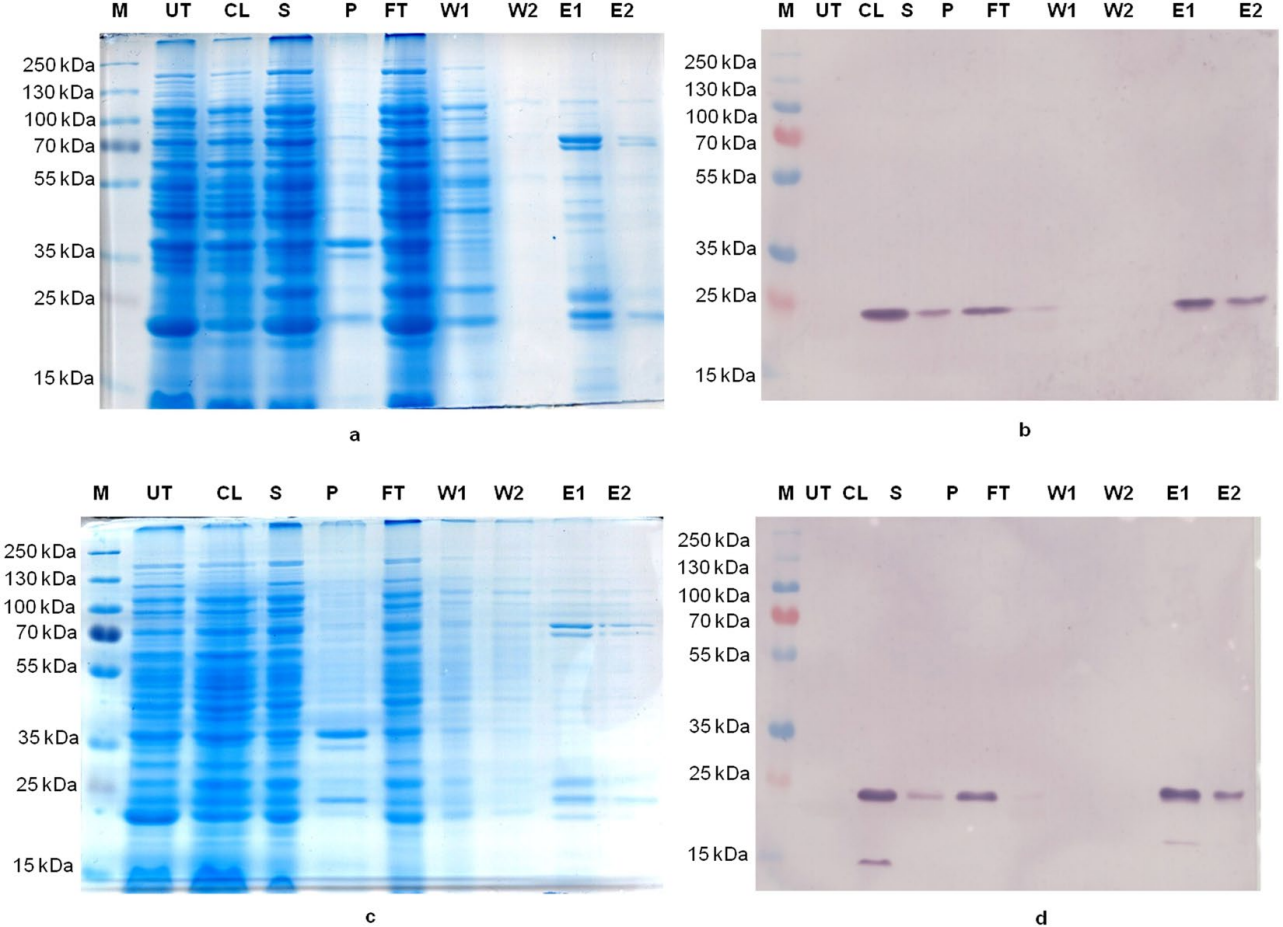
Small scale protein purification of LusPLA_2_s by Ni-NTA purification column by affinity chromatography. (**a**) SDS-PAGE analysis of the protein fractions of pET301/CT-DEST harbouring LusPLA_2_I-6xHis fusion protein. (**b**) Detection LusPLA_2_I-6xHis fusion protein by western blotting using anti-His antibodies. (**c**) SDS-PAGE analysis of the protein fractions of pET301/CT-DEST harbouring LusPLA_2_II-6xHis fusion protein. (**d**) Detection of LusPLA_2_II-6xHis fusion protein by western blotting using anti-His antibodies. M - Pageruler plus prestained protein ladder, UT-Untransformed BL21-RIPL, CL-crude lysate of transformed BL21-RIPL, S-supernatent, P-pellet, FT-flow through, W1-first wash, W2- second wash, E1- first elute and E2- second elute.

In order to assay the biochemical properties of cloned sPLA_2_s, both the proteins were expressed and purified as 6xHis-tagged fusion proteins. Both the genes, *LusPLA*_2_ *I* (502 bp) and *LusPLA*_2_ *II* (436 bp), were cloned in pENTR/SD/D/TOPO (see Supplementary Figs S2, S3) and mobilized into destination vector pET301/CT-DEST (see Supplementary Figs S4, S5). The successful cloning was confirmed by restriction of pET301/CT-DEST harbouring *LusPLA*_2_ *I-*6x-His with *Bam*HI and *Nco*I that released a fragment of ~574 bp (see Supplementary Fig. S4) and pET301/CT-DEST harbouring *LusPLA*_2_ *II-*6x-His with *Bam*HI and *Not*I that resulted in a fragment of 544 bp (see Supplementary Fig. S5). Sequencing of the expression clones confirmed that both the genes (*LusPLA*_2_ *I* and *LusPLA*_2_ *II*) were cloned in-frame with downstream 6x-His tag. Both the fusion proteins were obtained by induction (with 0.4 mM IPTG) in BL21-CodonPlus (DE3)-RIPL *E. coli* cells harbouring *LusPLA*_2_ *I* (Fig. 2a) and *LusPLA*_2_ *II* (Fig. 2c). Recombinant proteins were purified from the soluble fraction. Presence of an intense band of 22.6 kDa for PLA_2_I and 20.6 kDa for PLA_2_II was observed in SDS-PAGE. For PLA_2_ activity assay, both the proteins were purified from soluble fraction by affinity chromatography (Ni-NTA column). Precise detection of both the PLA_2_s were accomplished by western blotting that showed expected molecular weight of LusPLA_2_I as 22.6 kDa (Fig. 2b) and for LusPLA_2_II as 20.6 kDa (Fig. 2d).

### PLA_2_ activity assay

**Figure 3:**
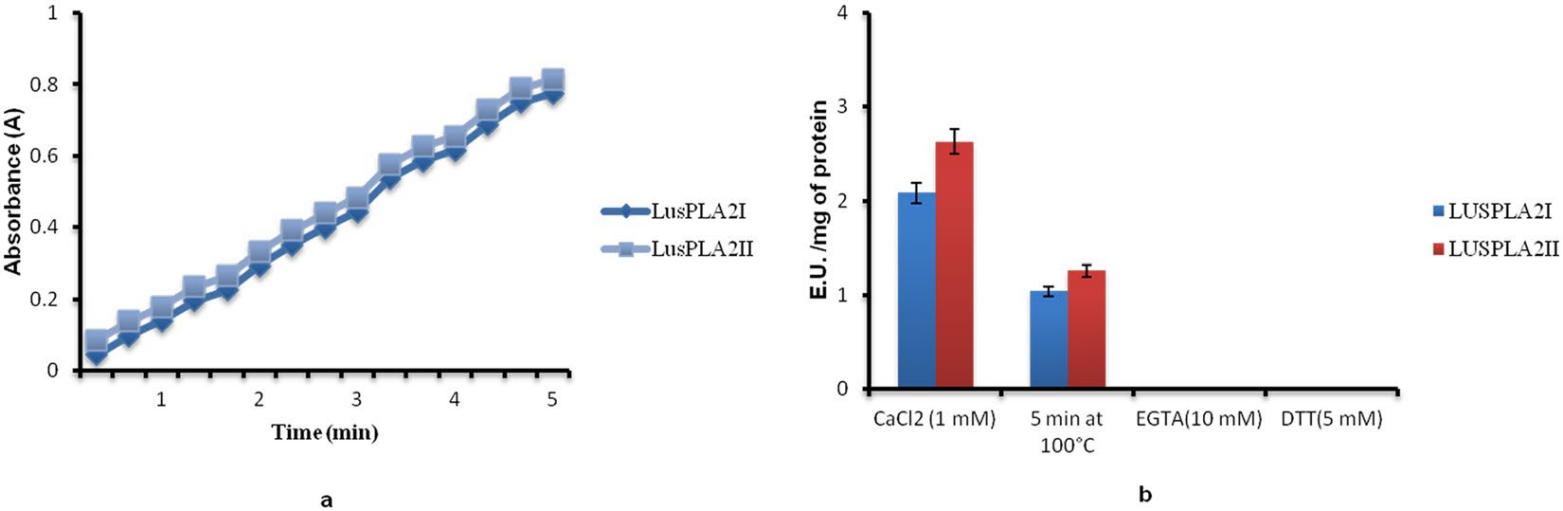
PLA_2_ activity and biochemical features of recombinant LusPLA_2_s fusion proteins. (**a**) PLA_2_ activity measured as increase in absorbance at 234 nm. The absorption was recorded every 20 seconds for 5 minutes. (**b**) Effect of ca^2+^ chelator (EGTA), disulphide bond destabilizer (DTT) and heat inactivation on PLA_2_ activity of recombinant protein. Data are expressed as mean value ± SD (3 independent experiments).

Purified PLA_2_s obtained through column chromatography were used to assess intrinsic lipase activity. The spectrophotometric assay was used to investigate the catalytic (hydrolysis of phospholipids) ability of the isolated LusPLA_2_I and LusPLA_2_II proteins. An increase in absorbance at 234 nm upon addition of phospholipases to substrate confirmed the isolated proteins as *bona fide* phospholipase enzymes (Fig. 3a). Thermostability of proteins was assessed by adding heat attenuated recombinant protein to the assay and was observed that LusPLA_2_I retained ~50% activity while LusPLA_2_II retained 48% of its activity (Fig. 3b). Similarly, effect of addition of divalent cations (Ca^2+^), chelating agents (EGTA) and reducing agents (DTT) on phopholipase activity of flax sPLA_2_s was assessed by adding CaCl_2_, EGTA, DTT. While, addition of divalent cation (Ca^2+^) chelator EGTA (10 mM) to the reaction completely attenuated the enzyme activity (Fig. 3b) of both sPLA_2_s, addition of disulphide reducing agent DTT (5 mM) to the reaction abolished the enzyme activity (Fig. 3b). However, addition of divalent cation CaCl_2_ restored the enzymatic properties of both flax sPLA_2_s (Fig. 3b).

### Physiochemical Properties

The relationship between molecular structure, functional properties and host of operating interaction accounts for the stability and biological activity of a protein. Analysis of physiochemical properties (see Supplementary Table S2) of flax sPLA_2_I and sPLA_2_II revealed the calculated molecular mass of LusPLA_2_I is 17.94 kDa and LusPLA_2_II is 15.72 kDa. The observed isoelectric point (pI) of LusPLA_2_I was 6.68 (pI < 7) and LusPLA_2_II was 8.84 (pI > 7). LusPLA_2_I was found to abundantly contain leucine followed by serine, cysteine, glycine and valine and deficient in phenylalanine and tryptophan. Likewise, glycine was most abundant amino acid in LusPLA_2_II followed by lysine, serine, leucine, cysteine and phenylalanine whereas tryptophan was least abundant (see Supplementary Table S3).

Stability of LusPLA_2_I and LusPLA_2_II was determined by calculating instability index (II)^21^. The instability index for LusPLA_2_I was 34.33 and for LusPLA_2_II was 17.13. Similarly aliphatic index (AI) is another parameter that is positively correlated with the thermal stability of globular proteins^22^. AI of LusPLA_2_I was found to be 95.72 and was classified as thermally more stable than LusPLA_2_II having AI of 69.79. Analysis of the Grand Average Hydropathy (GRAVY)^23^ revealed LusPLA_2_I (0.110) to be hydrophobic protein while LusPLA_2_II with GRAVY score of (-) 0.101 was classified as a hydrophilic protein (see Supplementary Table S2).

### Structural Analysis

Understanding of protein structure is of paramount importance for defining its precise function. Apart from the presence of conserved domains and motifs, the three dimensional structural similarity among sPLA_2_s from different groups is described solely in groups I-III and group X^17^ and are found to contain similar structural motifs. However, the structural data for group XI to which flax sPLA_2_ belong have not been studied in detail. Thus, we analyzed the secondary structure of both the flax sPLA_2_ proteins using predict protein software for occurence of alpha helix, extended strands and loops. The results classified LusPLA_2_I as “All alpha” type since it contained 49.40% alpha helix (>45% H)^24^ and LusPLA_2_II as “Mixed” type that contained 43.75% alpha helix (<45% H)^24^ (see Supplementary Fig. S6). Analysis of solvent accessibility composition revealed that both LusPLA_2_I and LusPLA_2_II are exposed type proteins with 62.65% and 61.81% residues on exposed surface respectively.

**Figure 4:**
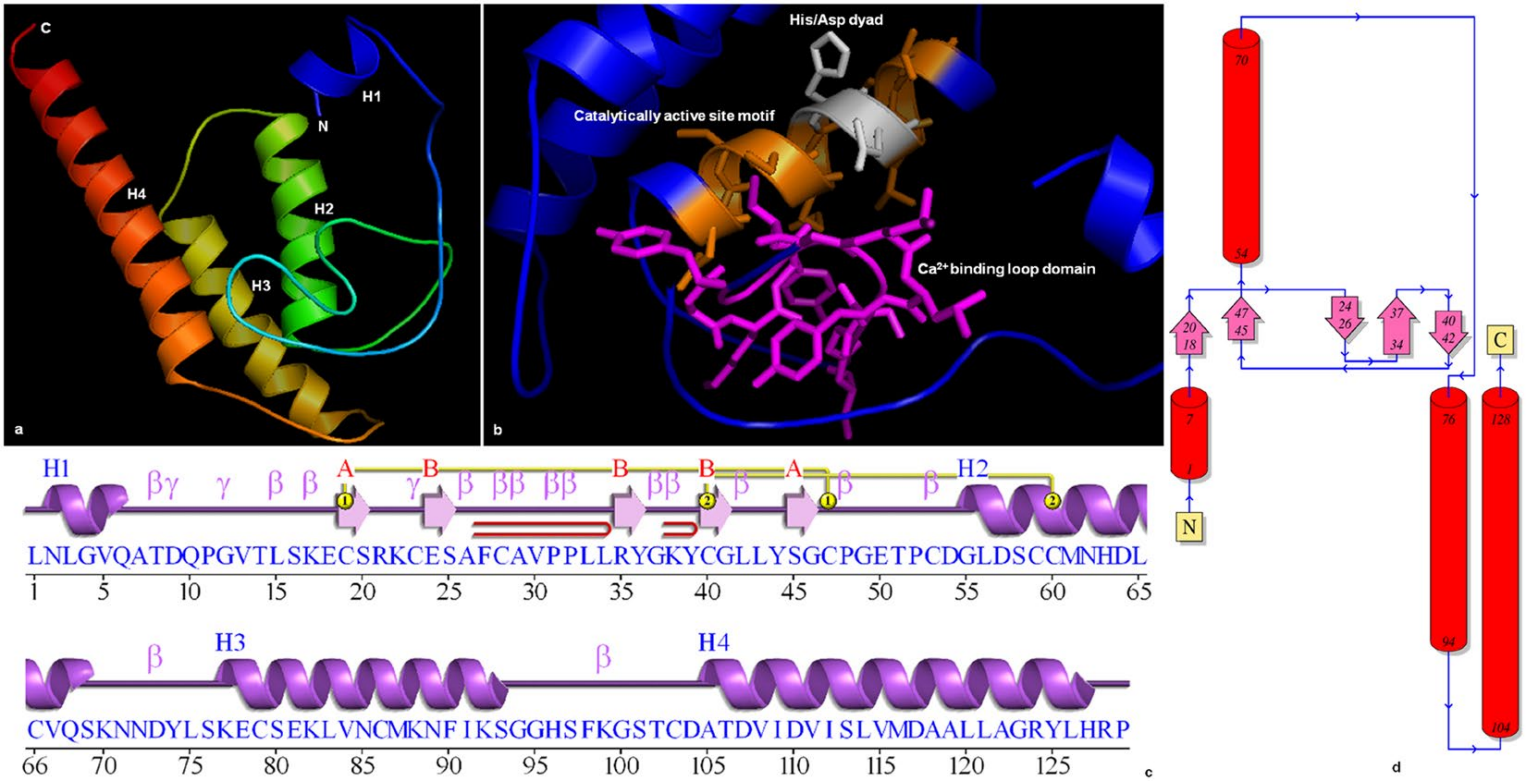
Structure of sPLA_2_I from flax. (**a**) Stereo ribbon diagram of the LusPLA_2_I monomer (chain *A*) color-coded from the N-terminus (blue) to the C-terminus (red). Helices (H1–H4) are indicated. (**b**) Ribbon diagram showing the conserved domains of sPLA_2._ Calcium binding loop is marked in pink, catalytically active site motif is marked in orange and conserved His/Asp dyad in gray. (**c**) Diagram showing the secondary-structure elements of LusPLA_2_I superimposed on its primary sequence. The labelling of secondary-structure elements is in accordance with *PDBsum* (http://www.ebi.ac.uk/pdbsum): α-helices are labeled H1–H4, five β-strands shown as arrow are labeled as A and B, β-turns and γ-turns are designated by their respective Greek letters (β, γ) and red loops indicate β-hairpins. (**d**) Topology of LusPLA_2_I protein showing the orientation of α-helices and β-strands.

**Figure 5:**
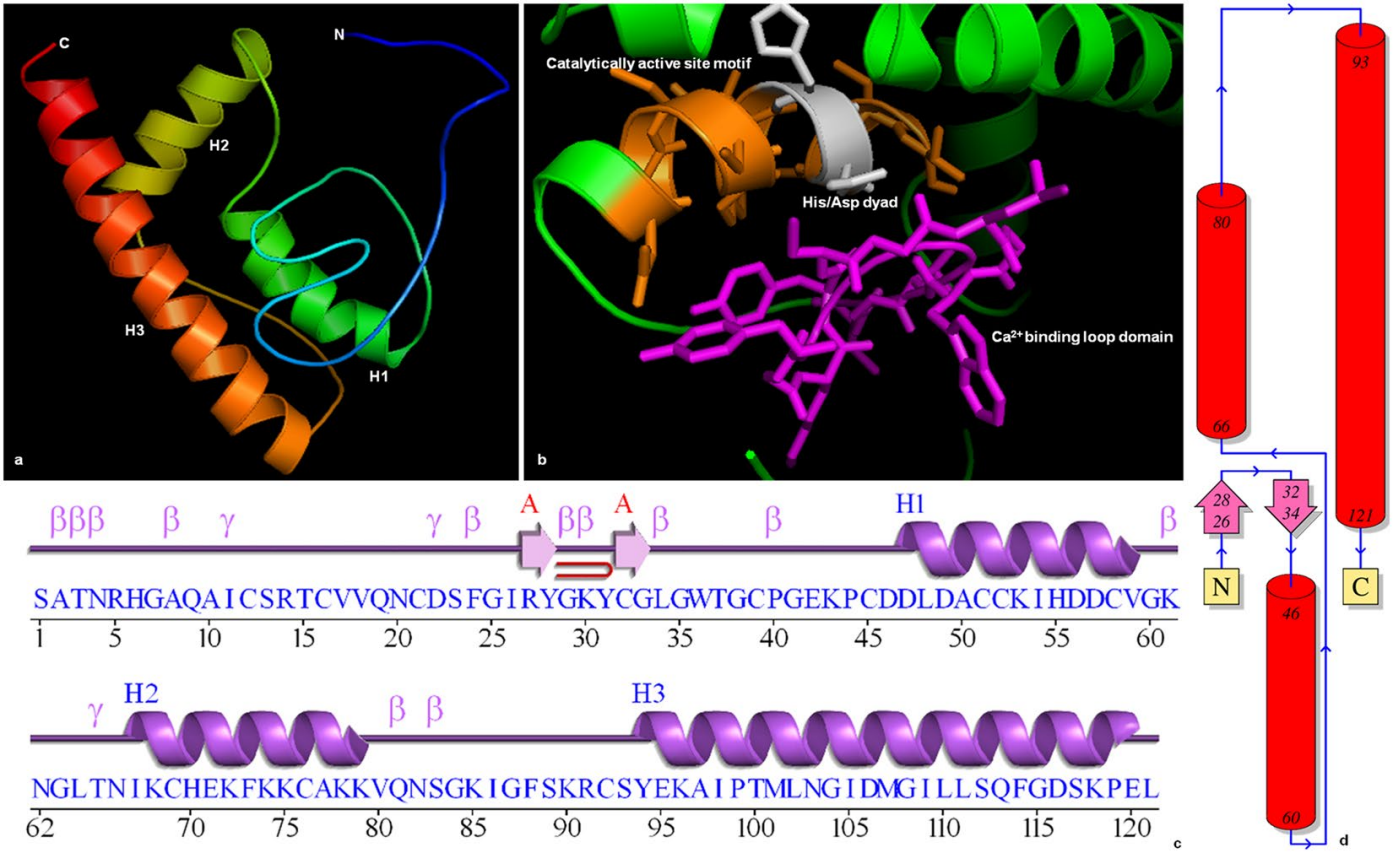
Structure of sPLA_2_II from flax. (**a**) Stereo ribbon diagram of the LusPLA_2_II monomer (chain *A*) color-coded from the N-terminus (blue) to the C-terminus (red). Helices (H1–H3) are indicated. (**b**) Ribbon diagram showing the conserved domains of sPLA_2._ Calcium binding loop is marked in pink, catalytically active site motif is marked in orange and conserved His/Asp dyad in gray. (**c**) Diagram showing the secondary-structure elements of LusPLA_2_II superimposed on its primary sequence. The labelling of secondary-structure elements is in accordance with *PDBsum* (http://www.ebi.ac.uk/pdbsum): α-helices are labeled H1-H3, two β-strands marked as arrow are labeled as A and B, β-turns and γ-turns are designated by their respective Greek letters (β, γ) and red loops indicate β-hairpins. (**d**) Topology of LusPLA_2_II protein showing the orientation of α-helices and β-strands.

Although three-dimensional structures of plant sPLA_2_s^18^ have been predicted, structural data for flax is unavailable. The homology modelled structures of LusPLA_2_I (Fig. 4a,b) and LusPLA_2_II (Fig. 5a,b) were generated using crystal structure of rice sPLA_2_II^18^ (Group-XIB) as template. While, LusPLA_2_I model had a C-score of 0.75, LusPLA_2_II had a C-score of 0.83. Additionally, the TM-score of 0.81 ± 0.09 and 0.83 ± 0.08 for LusPLA_2_I and LusPLA_2_II respectively confirmed the predicted model with correct topology. Analysis of stereo-chemical quality and accuracy of refined protein model using PROCHECK^25^ revealed that dihedral angles of all the residues were located in the most favoured regions (LusPLA_2_I- 91.0%; LusPLA_2_II- 90.1%) while 9.0% residues in LusPLA_2_I and 9.9% in LusPLA_2_II were present in additionally allowed region of the Ramachandran Plot (see Supplementary Figs S7, S8).

**Figure 6:**
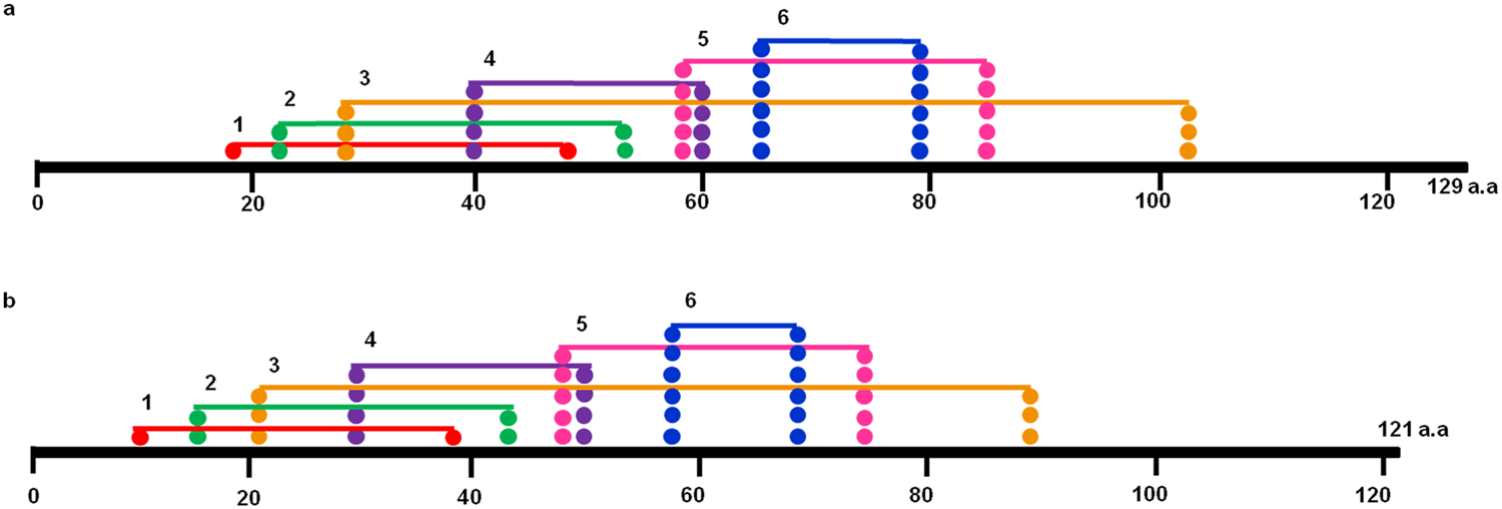
Distribution of six disulphide bridges in sPLA_2_s in flax. (**a**) In LusPLA_2_I all 12 Cys residues are involved in disulphide bond formation. Six potential disulphide bonds are formed between Cys56-Cys84, Cys60- Cys90, Cys65- Cys140, Cys77- Cys97, Cys96- Cys123 and Cys103- Cys116. (**b**) In LusPLA_2_II all 12 Cys residues are involved in disulphide bond formation. Six potential disulphide bonds are formed between Cys35-Cys62, Cys39- Cys68, Cys44- Cys115, Cys55- Cys75, Cys74- Cys99 and Cys81- Cys92.

The model is consistent with the secondary structure predictions of PDB sum^26^. Structure of LusPLA_2_I protein revealed presence of two β- sheets surrounded by four α-helices with one helix on left side and three on right side (Fig. 4c,d). The two β- sheets contained five β-strands out of which β- sheet A contained two parallel β-strands with topology 1X and β- sheet B contained three anti-parallel β-strands with topology 1 1 (Richardson nomenclature)^27^. The five beta strands are arranged in space as β-strands 1 (Cys19-Ser20), β-strands 3 (Arg35-Gly37) and β-strands 5 (Ser45-Gly46) in the same orientation and β-strands 2 (Glu24-Ala26) and β-strands 4 (Cys40-Gly41) in the opposite orientation. The protein structure also comprised of two β hairpins, fifteen β turns^28^ (see supplementary Table S4) and three γ turns. Of the four helices, α1 comprising Asn2-Gln6 (5 residues) surrounded the beta sheets on left side along with 15 residues of α2 (Gly55-Ser69), 17 residues of α3 (Lys77-Ser93) and 23 residues of α4 (Ala105-His127) on the right side. The two β-hairpins are incorporated in between β-strand 2–3 and 3–4 belonging to class^29^ 8:8 and 3:3 respectively surrounded by α1 (Asn2-Gln6) and α2 (Gly55-Ser69). The γ turns are of inverse type^30, 31^. All the twelve Cys residues that form disulfide bonds were harboured between Cys56-Cys84, Cys60- Cys90, Cys65- Cys140, Cys77- Cys97, Cys96- Cys123 and Cys103- Cys116 (Fig. 6a). Disulphide bonds formed between Cys56-Cys84, Cys60- Cys90 and Cys65- Cys140 connect the N- and C- terminal part of the protein, Cys77- Cys97 anchors the Ca^2+^ binding loop to α -helix 2; Cys96- Cys123 and Cys103- Cys116 tether the α-helix 2 to α-helix 3. The four α helices comprised of 60 residue (46.5%) whereas the β sheet comprised of 12 (9.3%) residues in LusPLA_2_I in flax.

Structure of LusPLA_2_II revealed presence of single β- sheet and three α-helices (Fig. 5c,d). The single β- sheet contained two anti-parallel β-strands (topology 1) that were arranged in space as β-strand 1 (Arg27-Tyr28) and β-strand 2 (Cys32-Gly33) in the opposite orientation. The structure comprised one β hairpins, thirteen β turns (see supplementary Table S5) and two γ turns. Major part of the protein is composed of three alpha helices such as 13 residues of α1 (Asp47-Val59), 13 residues of α2 (Ile67-Lys79) and 27 residues of α3 (Tyr94-Glu120). These helices start after the beta sheet covering the C-terminal portion of the protein. Only one β-hairpin was present between the strand 1–2 that belongs to class 3:3^29^. All the Cys residues that formed disulfide bonds were harboured between Cys35-Cys62, Cys39- Cys68, Cys44- Cys115, Cys55- Cys75, Cys74- Cys99 and Cys81- Cys92 (Fig. 6b) and stabilized the structure of protein. Disulphide bonds formed between Cys35-Cys62, Cys39- Cys68 and Cys44- Cys115 connect N- terminal to C- terminal portions of the protein; Cys55- Cys75 anchor Ca^2+^ binding loop to α -helix 1 and Cys74- Cys99 and Cys81- Cys92 tether α -helix 1 to α-helix 2. Three α helices comprised 53 residues (43.8%) whereas the β sheet comprised of four (3.3%) residues in LusPLA_2_ II in flax.

**Figure 7:**
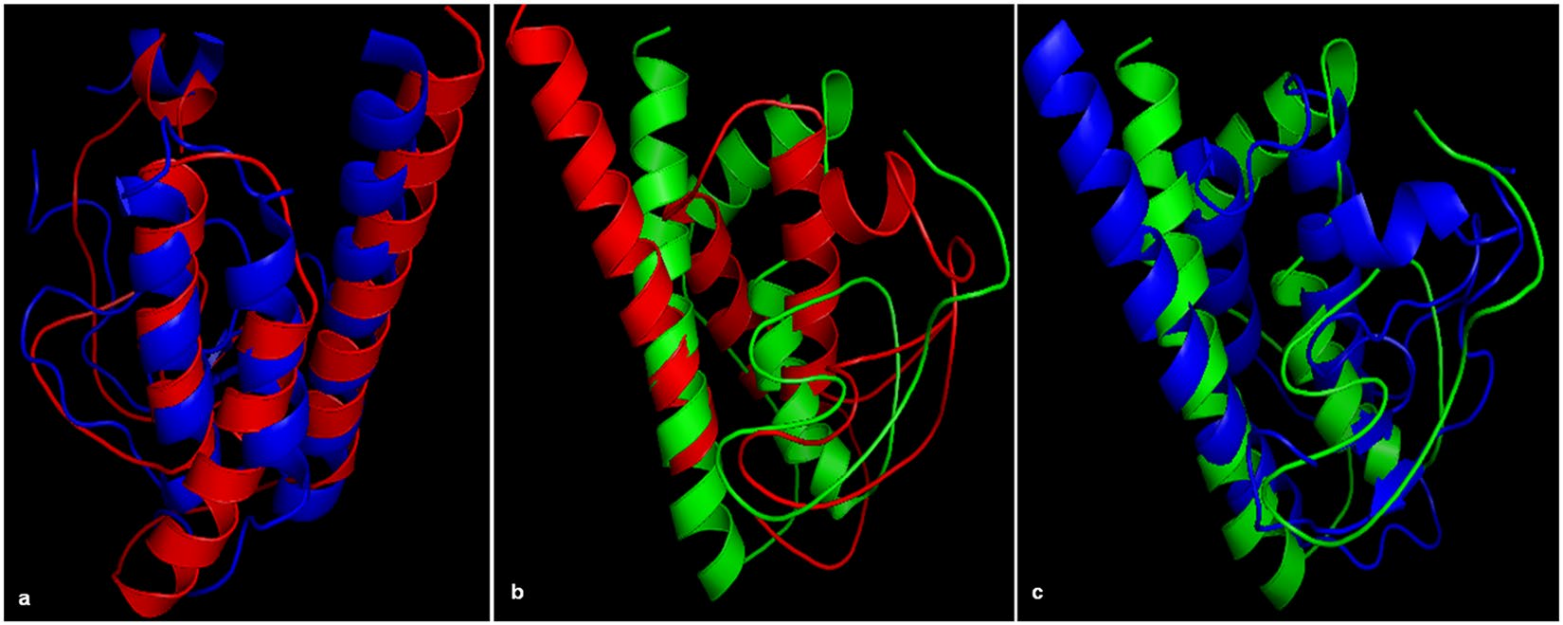
Comparision of tertiary structure of LusPLA_2_s among themselves and with OssPLA_2_ (2WG7A). The superimposed structures of OssPLA_2_ (2WG7A)-LusPLA_2_I and OssPLA_2_ (2WG7A)-LusPLA_2_II are similar in the calcium binding loop region and alpha helices. Structure of OssPLA_2_ shows more identitity to LusPLA_2_I. Although LusPLA_2_I contains 4 α- helices and LusPLA_2_II 3 α- helices, the superimposed isoform I and II of flax sPLA_2_ are also similar in the conserved calcium binding loop and catalytically active site (**a**) Superimposed structure of OssPLA_2_ (2WG7A) – LusPLA_2_I. (**b**) Superimposed structure of OssPLA_2_ (2WG7A) – LusPLA_2_II. (**c**) Superimposed structure of LusPLA_2_I- LusPLA_2_II. Cartoon diagram in red indicates the 3-D structure of OssPLA_2_ (2WG7A), Cartoon diagram in blue indicates the 3-D model of LusPLA_2_I and Cartoon diagram in green indicates the 3-D model of LusPLA_2_II.

The superimposed structures of OssPLA_2_ (2WG7A) -LusPLA_2_I (Fig. 7a) and OssPLA_2_ (2WG7A) -LusPLA_2_II (Fig. 7b) exhibited homology in the calcium binding region and alpha helices. Superimposed isoform I and II of flax sPLA_2_ (Fig. 7c) were also found to be similar in the calcium binding loop and alpha helices.

## Discussion

Flax entered genomics research lately with decoding of its genome sequence in 2014^32^. Subsequently, genomic information were generated for abiotic stress tolerance in flax^33–36^. Flax is utilized as a multipurpose crop with industrial as well as pharmaceutical use. It yields three economically important products viz. seed oil that is rich in omega-3-fatty acids, bast fiber i.e. linen and nutraceuticals. While, flax seed oil have industrial applications nutraceuticals are used in food industry. Among nutraceuticals, utility of phospholipases from other organisms have been reported in food industry as emulsifiers and degumming agents^4^. However, phospholipase from plant source have not been explored for use in food industry.

In our endeavour, we identified two flax sPLA_2_s in its genome (LusPLA_2_I and LusPLA_2_II) on the basis of their homology in protein sequence, domain structure and phylogenetic relationship (see Supplementary Fig. S1) with other known plant sPLA_2_s^37^. A single phospholipase A_2_ signature (PA2c) domain with ID cd04706 was identified in both the proteins (see Supplementary Table S1). Secretory PLA_2_s containing cd04706 have been identified from many plants including rice^18^. This domain contained a conserved Ca^2+^ binding loop and a catalytic domain with enzymatically active His/Asp dyad. His residue of His/Asp dyad is involved in the deprotonation of ester carbonyl carbon of the substrate and Asp residue interacts with Ca^2+^ cofactor through its β carbonyl group^6^. Conserved residues involved in hydrogen bonding in animals, are found in the Ca^2+^ binding loop of plant phospholipases^38^. Pairwise alignment of flax sPLA_2_s with plant sPLA_2_ revealed that all conserved motifs identified in arabidopsis and rice^8^ are present in flax. However, minor variations in conserved moieties were observed such as replacement of conserved Asp moiety by His93 in LusPLA_2_II and by Ser117 in LusPLA_2_I. Similar, replacement of conserved Ala by Ser residue in catalytic domain has been reported in arabidopsis sPLA_2_α and citrus^8, 39^. Nevertheless, the impact of this replacement on the catalytic activity of enzyme is yet to be elucidated.

*In-silico* tools predicted LusPLA_2_II to be secreted into the extracellular space (see Supplementary Table S6). Our result is in accordance with arabidopsis (AtsPLA_2_β and AtsPLA_2_γ) and rice sPLA_2_ isoforms (OssPLA_2_β and OsPLA_2_γ)^14, 40^ that have been confirmed to be secreted into extracellular space. While, LusPLA_2_II was localized into extracellular space, LusPLA_2_I is predicted to be secreted into the mitochondria as well as extracellular space (see Supplementary Table S6). This is commensurate with the detection of sPLA_2_ activity in mitochondria of durum wheat^41^. Since AtsPLA_2_α and OssPLA_2_I are localized into Golgi bodies and ER^14^, we speculate LusPLA_2_I is secreted into the extracellular space and re-localized to mitochondria or vice-versa on interaction with other proteins or in response to external stimuli. Nuclear re-localization of AtsPLA_2_α upon interaction with *AtMYB30* ^42^ also supports our finding. This is plausible, owing to the plethora of roles played by sPLA_2_ in various cellular processes.

Ontological classification of flax sPLA_2_s revealed their involvement in many biological and metabolic processes such as growth and development, lipid catabolic process, auxin response, gravitropism, guard cell movement^43^, and root development^44^ (Table 1). Several studies have highlighted the involvement of sPLA_2_ in wounding and pathogen attack^45^, cold and salinity tolerance^46^. Recently several isoforms of sPLA_2_s were reported to be highly up-regulated during water stress in wheat^15^, rice^14^ and arabidopsis^46^. In wheat, PLA_2_ activity concomitantly increases during drought stress^15^ while sPLA_2_s have been implicated in hyperosmotic stress in *Chlamydomonas* ^47^.

Biochemical characterization of both LusPLA_2_ proteins revealed them to be *bona fide* phospholipase A_2_. Detailed molecular analysis by SDS-PAGE and western blot analysis revealed that observed molecular weight of recombinant proteins were ~22.6 kDa and ~20.6 kDa for LusPLA_2_I (Fig. 2a,b) and LusPLA_2_II (Fig. 2c,d) respectively. The observed molecular weight, after accounting for 6x-his tag (~4.7 kDa), of LusPLA_2_I (~17.9 kDa) and LusPLA_2_II (~15.7 kDa) respectively is in congruence with their predicted molecular weight. PLA_2_/LOX- coupled spectrophotometric assay of both the proteins revealed that cloned sPLA_2_s of flax are *bona fide* lipases as the increase in absorbance results from the release of free linoleate from PC_LIN_ by the activity of LusPLA_2_I and LusPLA_2_II (Fig. 3a). Detailed, enzymatic assay of both proteins revealed requirement of micro to mili molar concentration of calcium for their optimum activity. Complete abolition of enzymatic activity by addition of 10 mM EGTA, a potent Ca^2+^ chelator, confirmed our observation (Fig. 3b). The inhibition of enzyme activity due to unavailability of Ca^2+^ can be ascribed to destabilization of transition-state intermediate^48^. Similarly, addition of 5 mM DTT, a disulphide bond reducing agent, completely prevented the protein activity (Fig. 3b). This can be attributed to the destabilization of intra-molecular disulphide bridges^17^ prevalent in LusPLA_2_I and LusPLA_2_II protein. Similarly, thermostability assay of both the recombinant proteins viz. LusPLA_2_I and LusPLA_2_II revealed 50% of their enzymatic activity is retained after boiling at 100°C for 5 minutes (Fig. 3b). It has been reported that wheat sPLA_2_ isoform III retains ~45% of enzyme activity after boiling at 100°C for 5 minutes^49^. We belive structural stability provided by six disulphide bridges account for the thermo-stability of the proteins.

Analysis of physiochemical properties of both the proteins revealed they have different pI values for LusPLA_2_I (6.68) and LusPLA_2_II (8.84) (see Supplementary Table S2). The difference in pI makes LusPLA_2_I to be acidic while LusPLA_2_II to be basic. Acidic sPLA_2_s are also reported from rice (isoform I and III) and soybean-XIA-1 where as slightly alkaline sPLA_2_s are reported from arabidopsis and soybean-XIA-2^16^. Neutral sPLA_2_s are reported from carnation (*Dianthus caryophyllus*) and tomato (*Solanum lycopersicum*)^16^. The amino acid composition of both proteins revealed that LusPLA_2_I is rich in hydrophobic amino acids while LusPLA_2_II is abundant in hydrophilic amino acid followed by charged amino acid lysine (see Supplementary Table S3). Another parameter, protein instability index of both proteins revealed them to be highly stable. Usually, proteins with instability index values greater than 40 are considered to be unstable proteins^21^. Both LusPLA_2_s were found to be highly stable as indicated by instability index value below 40 (LusPLA_2_I- 34.33 and LusPLA_2_II- 17.13) and higher aliphatic index (LusPLA_2_I- 95.72 and LusPLA_2_II- 60.79) (see Supplementary Table S2). Among the two, LusPLA_2_I is more stable than LusPLA_2_II. The lower stability of LusPLA_2_II is indicative of structural flexibility. Additionally, structural flexibility of LusPLA_2_II can also be ascribed to abundance of glycine moieties that are generally found at the surface of the proteins, within the loops or coils and provide flexibility. Grand average hydropathy results revealed that LusPLA_2_I with GRAVY score of 0.110 is hydrophobic and LusPLA_2_II with GRAVY score of (-) 0.101 is hydrophilic (see Supplementary Table S2). A negative GRAVY score indicates soluble nature of the protein. Abundance of glycine with negative GRAVY score further explains the hydrophilic nature of LusPLA_2_II.

Insight into the secondary structure of flax sPLA_2_s indicated that they are dominated by alpha helices followed by random coils (see Supplementary Fig. S6). This is corroborated by the three-dimensional model that revealed the presence of 4 α-helices in LusPLA_2_I (Fig. 4) and 3 *α*-helices in LusPLA_2_II (Fig. 5). The precise function of a protein depends on interaction of its exposed surface with solvent. It was found that both the proteins contain more than 60% residues exposed on the surface. This property might account for the involvement of sPLA_2_s in host of biological processes. Our result suggests, the three-dimensional model of both sPLA_2_s of flax generated by I-TASSER^50, 51^ was of correct topology due to higher C-score value of the predicted model. The C-score is a measure of quality of the predicted model and its value ranges from (-) 5 to 2. A higher C-score indicates a high quality model and C-score > (-) 1.5 indicates the correct folding^52^. The C-score values of 0.75 and 0.83 obtained for LusPLA_2_I and LusPLA_2_II respectively indicate that both the protein models are of good quality and correct folding.

Similarly, the TM-score > 0.5 indicates a model of correct topology and a TM-score < 0.17 means random similarity. The desired TM score of 0.81 ± 0.09 and 0.83 ± 0.08 for LusPLA_2_I and LusPLA_2_II respectively indicate the correct topology of modelled protein (Figs 4a and 5a). These results were further corroborated by the stereochemical stability assessment by Ramachandran plot analysis (see Supplementary Figs S6, S7). Occurrence of 90% or more residues in the most favoured region of Ramachandran plot, classified the refined models of LusPLA_2_ proteins to be of good quality (see Supplementary Figs S6, S7). The arrangement of 6 disulphide bonds formed by 12 conserved Cys residues is also in accordance with known plant sPLA_2_s (Fig. 6a,b)^16^.

Both the models were based on principle template crystal structure of *Oryza sativa* sPLA_2_II (2WG7A)^18^. Despite the deviations in primary and secondary structures, the tertiary structure of flax sPLA_2_s is well conserved in the regions essential for precise function of the enzyme viz, Ca^2+^ binding loop and catalytic active site containing conserved His/Asp dyad (Figs 4b,c,d and 5b,c,d). This was commensurate with the superimposed structures of OssPLA_2_-LusPLA_2_I, OssPLA_2_-LusPLA_2_II and LusPLA_2_I-LusPLA_2_II (Fig. 7). Superimposed structure of OssPLA_2_-LusPLA_2_I revealed that they were similar and both OssPLA_2_ and LusPLA_2_I contained four α-helices. While Ca^2+^ binding loop did not cover α-helix, catalytically active site motif covered most of the region of α-helix 2 in both the proteins. LusPLA_2_II contained only three α-helices. The α-helix covering the N-terminal end of OssPLA_2_ was missing in LusPLA_2_II. In LusPLA_2_II also, Ca^2+^ binding loop did not cover α-helix and catalytically active site motif covered most of the region of α-helix 2. However, the connecting structures varied in their length and morphology among the two LusPLA_2_s and the template (Fig. 7).

In summary, our work provides first insight into the structure and catalytic mechanism of two sPLA_2_s in flax. In this endeavour, we expressed and purified active LusPLA_2_I and LusPLA_2_II from soluble fraction. We carried out the first biochemical characterization of sPLA_2_s from flax and provides insight into the mechanism of sPLA_2_ enzyme. The holomeric structure of both the proteins revealed that they are of high quality and topology. Such biochemical and structural analysis providing insight into the structure and function of an important class of protein is required for fine tuning of their *in planta* expression for improving performance of flax during stress.

## Methods

### Sequence Retrieval and analysis of sPLA_2_

Protein sequence of secretory phospholipase A_2_ of arabidopsis (*Arabidopsis thaliana*), rice (*Oryza sativa*), soybean (*Glycin max*), citrus (*Citrus sinensis*), wheat (*Triticum durum*), flax (*Linum usitatissimum*) and snake venom (*Naja kaouthia*) were retrieved from the sequence repository of the NCBI database (www.ncbi.nlm.nih.gov/) (see Supplementary Table S6). Snake venom sPLA_2_ sequence was included as an out-group in the study. The conserved domains specific to secretory phospholipase A_2_ were identified using the NCBI Conserved Domain Database^53^ CDD v3.14–47363 PSSMs (http://www.ncbi.nlm.nih.gov/Structure/cdd/wrpsb.cgi) annotations. The motifs were predicted using MEME suite^54^ (http://meme.nbcr.net/meme/cgi-bin/meme.cgi). The parameter of motif size was set at six amino acids as minimum and 50 amino acids as maximum. The data were analysed using SignalP for post-translational modifications^55^. Putative localization of flax sPLA_2_s were predicted by TargetP^56^ and iPSORT^57^. The sequences of proteins were aligned using Mafft-win^58^ and viewed using ESPRIPT^59^ and phylogenetic tree was constructed by maximum likelyhood method using MEGA6^60^. UniProt database (http://www.uniprot.org) was used to assign gene ontology classifications^61^.

### Cloning, expression and purification of sPLA_2_ protein

Total RNA isolated from flax leaves was used for cDNA synthesis using SuperScript™ III First-Strand Synthesis supermix following the manufacturer’s instructions (Life technologies Corporation). Sequence of flax sPLA_2_ isoform *LusPLA*_2_ *I* (Genbank accession: KU361324) and *LusPLA*_2_ *II* (Genbank accession: KU361324) were amplified using primer pairs listed in Supplementary Table S7. The amplified gene products were cloned in Gateway entry vector pENTR/SD/D/TOPO (Life technologies Corporation) as per manufacturer’s instructions and authenticity was confirmed by sequencing. For protein expression, the *LusPLA*_2_ *I* and *LusPLA*_2_ *II* genes were mobilized from entry vector into the destination vector, pET301/CT-DEST (Invitrogen Corporation) to generate the expression clones pET301/CT-DEST harbouring *LusPLA*_2_ *I*-6xHis and pET301/CT-DEST harbouring *LusPLA*_2_ *II*-6xHis as per manufacturer’s instructions.

The *E.coli* (CodonPlus (DE3)-RIPL cells (Agilent Technologies) cells harbouring the *LusPLA*_2_ *I*- 6xHis and *LusPLA*_2_ *II*-6xHis recombinant plasmid were grown at 37°C in LB medium containing 100 µg/ml carbenicillin until OD_600_ = 0.6 and induced with 0.4 mM IPTG (isopropyl-β-D-thiogalactopyranoside) for 4 h. The cells were harvested by centrifugation at 10,000 rpm, 4°C for 10 min and lysed by re-suspending in native lysis buffer containing lysozyme and sonicated at 24–25% amplitude 30 sec ON/OFF cycle on ice for 12–15 minutes ON condition. The recombinant proteins were purified by using Ni-NTA affinity chromatography by using QIAexpress® Ni-NTA Fast Start kit by following manufacturer’s instructions. The purity and yield of recombinant protein were analysed by SDS-PAGE and protein content was determined by Bradford’s method^62^.

### SDS-PAGE and western blotting

The recombinant fusion protein LusPLA_2_I-6xHis and LusPLA_2_II-6xHis were resolved on a 12% (w/v) SDS-PAGE as described earlier^63^ and visualized after coomassie brilliant blue staining. For western blot analysis, both the proteins were resolved on 12% acrylamide gel and transferred to PVDF (polyvinylidene diflouride) membrane using electro-blot system (Major science, USA) at constant voltage of 100 V, 15°C for 3 h in 1X Towbin buffer (25 mM Tris, 192 mM glycine, 20% methanol and 0.05% SDS). The western blotting was performed using WesternBreeze® Chemiluminescent Kit (Life technologies Corporation), as per manufacturer’s instructions.

### PLA_2_ activity determination

PLA_2_ activity was evaluated using spectrophotometric method based on PLA_2_/lipoxygenase (LOX) coupled reactions^15^. The PLA_2_ activity was assayed by addition of the recombinant protein to 4 E.U. LOX, 0.5 mM 1,2-diacyl-sn-glycero-3-phosphocholine (PC_LIN_) and 1 mM CaCl_2_ in 2 ml 50 mM sodium borate buffer (pH 9.0). Increase in absorbance at 234 nm was monitored. The effect of 10 mM ethylene glycol bis (-2 amino ethyl ether) – N, N, N, N – tetra acetic acid (EGTA), a Ca^2+^ chelator and 5 mM dithiothreitol (DTT), a disulphide bond reducing agent on enzyme activity was evaluated. Effect of heat (100°C for 5 min)^15^ on protein activity was studied to ascertain thermostability of proteins.

### Physiochemical characterisation and secondary structure analysis

To gather the structural information about flax sPLA_2_s, physiochemical properties and secondary structure was predicted for both the proteins. Physio-chemical characterization was performed using the Expasy’s ProtParam server (http://us.expasy.org/tools/protparam.html)^64^. Isoelectric point (pI), molecular weight, instability index^21^, aliphatic index^22^ and grand average hydropathy (GRAVY)^23^ were predicted to estimate the stability of the protein. Predict Protein server (https://www.predictprotein.org/)^65^ was employed for secondary structure prediction of both LusPLA_2_ isoforms. Presence of alpha helices, strands and loops were analyzed and exposed surface for solvent accessibility of protein was calculated by Predict Protein server.

### Homology modelling and evaluation

Multiple online tools are available for predicting the three dimensional model of protein such as Chunk-TASSER^66^, Meta-TASSER^67^, Pro-sp3-TASSER^68^, TASSER-VMT^69^ and I-TASSER^70^. Initial model of flax sPLA_2_I and sPLA_2_II was constructed using fully automated I-TASSER server (http://zhanglab.ccmb.med.umich.edu/I-TASSER/) that combined threading and *ab-initio* modelling for the prediction which automatically selected rice sPLA_2_II as the template for modelling. The initial model generated using I-TASSER was refined further by ModRefiner (http://zhanglab.ccmb.med.umich.edu/ModRefiner/). The refined models were checked for stereo-chemical properties by Ramachandran Plot analysis using RAMPAGE (http://mordred.bioc.cam.ac.uk/~rapper/rampage.php)^71^. The residues falling in the disallowed region of the Ramachandran Plot were further refined by Modloop, which automatically modelled the loops of protein structures (https://modbase.compbio.ucsf.edu/modloop/)^72^. The protein model was refined until all the amino acid residues fell in the favoured region of the Ramachandran plot (RAMPAGE). The validation of the modelled structure was performed to determine the accuracy of secondary structure predictions and modelling by PDBSum (https://www.ebi.ac.uk/pdbsum/)^26^. PROCHECK was used to confirm if all the residues were falling in the most favoured regions of the Ramachandran Plot^25^. Structure visualization was performed with pymol (http://www.pymol.org). The predicted models of proteins were submitted to Protein Model Database (PMDB)^73^ and have been assigned identifier PM0080416 (LusPLA_2_I) and PM0080415 (LusPLA_2_II).

### Data availability

All data generated or analysed during this study are included in this published article (and its Supplementary Information files).

## Electronic supplementary material

Supplementary Information
