# Supplementary material for: Molecular details of secretory phospholipase A2 from flax (Linum usitatissimum L.) provide insight into its structure and function
Source: Sci Rep. 2017 Sep 11;7:11080. doi: 10.1038/s41598-017-10969-9 (PMC5593939; doi:10.1038/s41598-017-10969-9)
Supplement: Supplementary file 1 — Supplementary Information [file 41598_2017_10969_MOESM1_ESM.pdf]

Supplementary information

Molecular details of secretory phospholipase A_2 from flax (*Linum usitatissimum* L.) provide insight into its structure and function.

Payal Gupta[1,2*] and Prasanta K Dash[1*] .

[1] ICAR-National Research Centre on Plant Biotechnology, Pusa Campus, New Delhi-110012, India.

[2] Department of Biotechnology, Kurukshetra University, Thanesar-136119, India.

*Correspondence: payalgupta33@gmail.com; pdas@nrcpb.org

Supplementary Figures

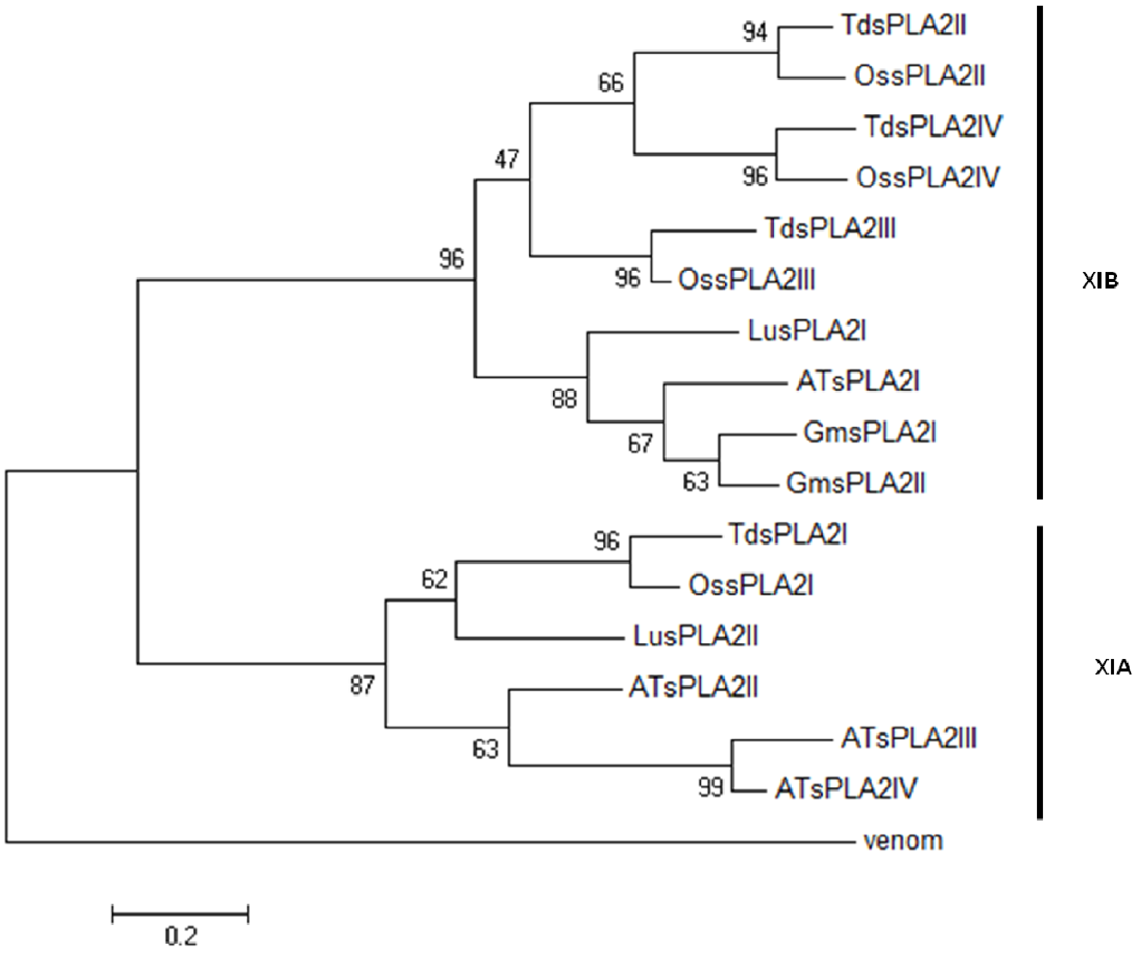

Figure S1: Phylogeny of plant sPLA$_2$s including of flax sPLA$_2$s (LusPLA$_2$I and LusPLA$_2$II). Phylogenetic tree of the deduced amino acid sequences of the two LusPLA$_2$s with known orthologous genes from *Arabidopsis*, rice and soybean to show evolutionary conservation among plants.

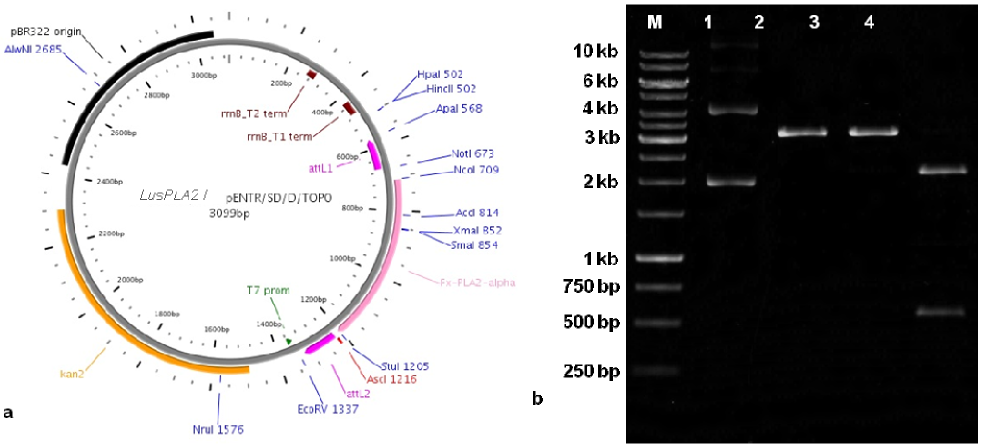

Figure S2: *LusPLA$_2$I* in pENTR/SD/D/TOPO generated by gateway cloning. (a) Vector map of *LusPLA$_2$I* in pENTR/SD/D/TOPO entry clone. (b) Confirmation of *LusPLA$_2$I* in pENTR/SD/D/TOPO by restriction digestion with *Not*I/*Asc*I. Lane M: 1 kb DNA ladder (Fermentas). Lane 1: uncut plasmid DNA. Lane 2: ~ 3kb fragment of *LusPLA$_2$I* in pENTR/SD/D/TOPO restricted with *Not*I. Lane 3: ~ 3kb fragment of *LusPLA$_2$I* in pENTR/SD/D/TOPO restricted with *Asc*I. Lane 4: Restriction digestion with *Not*I/*Asc*I released a fragment of ~500 bp of *LusPLA$_2$I* gene and ~2.6 kb fragment of pENTR/SD/D/TOPO vector backbone.

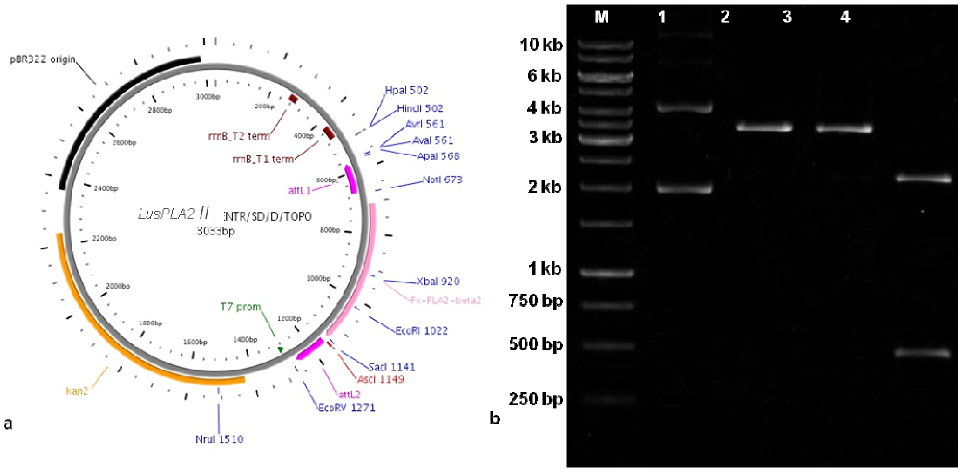

Figure S3: *LusPLA$_2$II* in pENTR/SD/D/TOPO generated by gateway cloning. (a) Vector map of *LusPLA$_2$II* in pENTR/SD/D/TOPO entry clone. (b) Confirmation of *LusPLA$_2$II* in pENTR/SD/D/TOPO by restriction digestion with *Not*I/*Asc*I. Lane M: 1 kb DNA ladder (Fermentas). Lane 1: uncut plasmid DNA. Lane 2: ~ 3kb fragment of *LusPLA$_2$II* in pENTR/SD/D/TOPO restricted with *Not*I. Lane 3: ~ 3kb fragment of *LusPLA$_2$II* in pENTR/SD/D/TOPO restricted with *Asc*I. Lane 4: Restriction digestion with *Not*I/*Asc*I released a fragment of ~430 bp of *LusPLA$_2$II* gene and ~2.6 kb fragment of pENTR/SD/D/TOPO vector backbone.

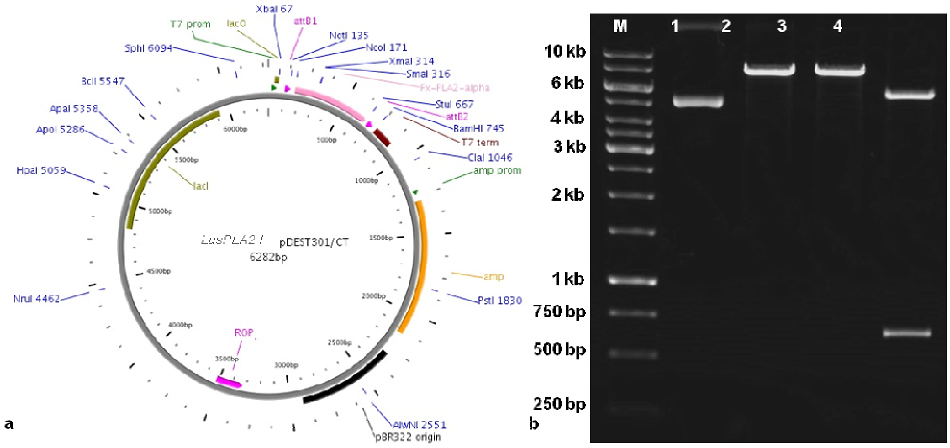

Figure S4: Cloning of *LusPLA₂I* in pET301/CT-DEST vector by gateway cloning. (a) Vector map of pET301/CT-DEST harbouring *LusPLA₂I*-6xHis destination clone. (b) Confirmation of pET301/CT-DEST harbouring *LusPLA₂I*-6xHis by restriction digestion with *Bam*HI/*Nco*I. Lane M: 1 kb DNA ladder (Fermentas). Lane 1: uncut plasmid DNA. Lane 2: ~ 6.2kb fragment of pET301/CT-DEST harbouring *LusPLA₂I*-6xHis restricted with *Bam*HI. Lane 3: ~ 6.2kb fragment of pET301/CT-DEST harbouring *LusPLA₂I*-6xHis restricted with *Nco*I. Lane 4: Restriction digestion with *Bam*HI/*Nco*I released a fragment of ~574 bp of *LusPLA₂I* gene and ~5.7 kb fragment of pET301/CT-DEST vector backbone.

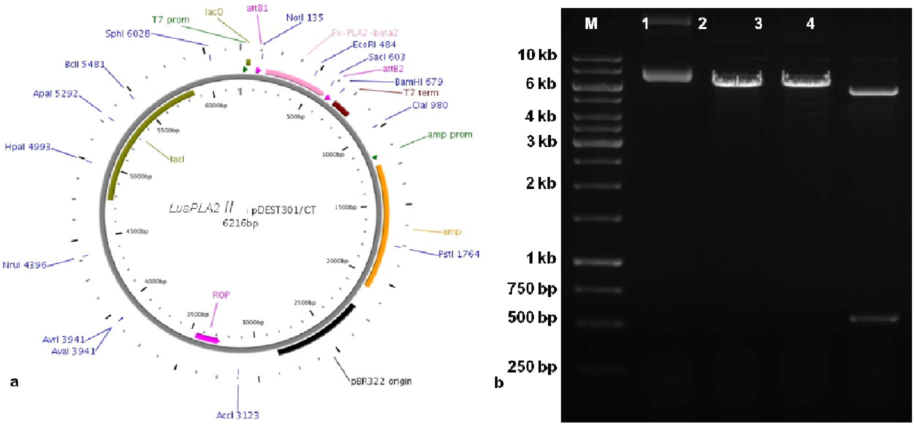

Figure S5: Cloning of *LusPLA$_2$II* in pET301/CT-DEST vector by gateway cloning. (a) Vector map of pET301/CT-DEST harbouring *LusPLA$_2$II*-6xHis destination clone. (b) Confirmation of pET301/CT-DEST harbouring *LusPLA$_2$II*-6xHis by restriction digestion with *Bam*HI/*Not*I. Lane M: 1 kb DNA ladder (Fermentas). Lane 1: uncut plasmid DNA. Lane 2: ~ 6.2kb fragment of pET301/CT-DEST harbouring *LusPLA$_2$II*-6xHis restricted with *Bam*HI. Lane 3: ~ 6.2kb fragment of pET301/CT-DEST harbouring *LusPLA$_2$II*-6xHis restricted with *Not*I. Lane 4: Restriction digestion with *Bam*HI/*Not*I released a fragment of ~544 bp of *LusPLA$_2$II* gene and ~5.6 kb fragment of pET301/CT-DEST vector backbone.

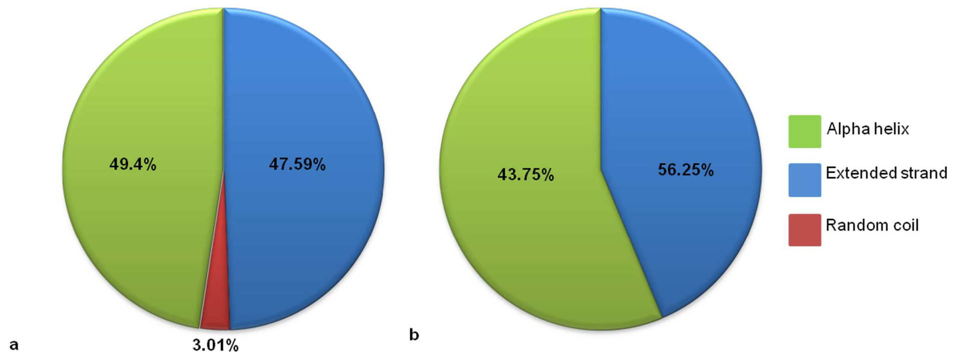

Figure S6: Structural analysis of flax sPLA$_2$s. (a) LusPLA$_2$I is classified as all alpha type containing 49.4% alpha helix (> 45% helix). (b) LusPLA$_2$II is classified as mixed type containing only 43.75% alpha helix (< 45% helix).

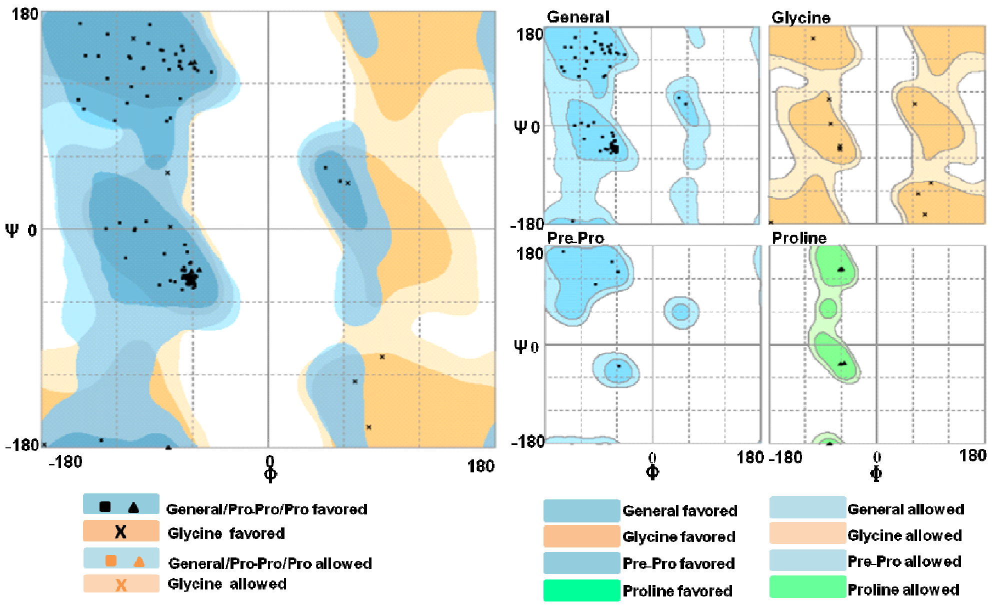

Figure S7: Stereo-chemical analysis of LusPLA2I by Ramachandran Plot. Stereo-chemical properties of LusPLA$_2$I was analysed by Ramachandran Plot using RAMPAGE with 91% residues in favoured region and 9% residues in the allowed region.

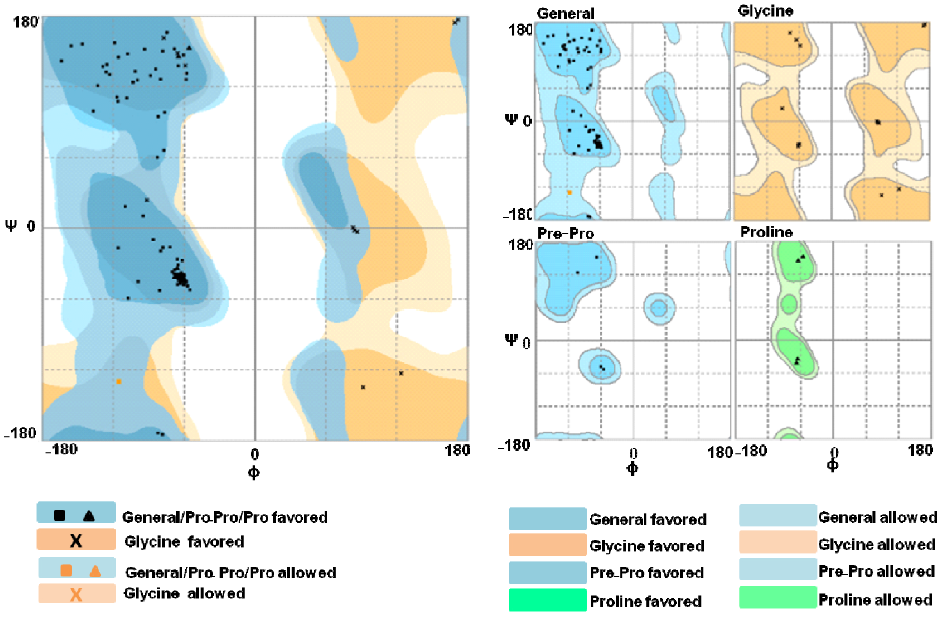

Figure S8: Stereo-chemical analysis of LusPLA$_2$II by Ramachandran Plot. Stereo-chemical properties of LusPLA$_2$II by Ramachandran Plot analysis using RAMPAGE with 90.1% residues in favoured region and 9.9 % residues in allowed region.

Supplementary Tables

Supplementary Table S1: Conserved domains identified in $LusPLA_2$s.

Proteins	Conserved Domains	Accession	Position	E-value
$LusPLA_2I$	$Plant\_PLA_2$	cd04706	50 - 166	3.24e-55
$LusPLA_2II$	PLA_2_like	cl05417	35-138	4.92e-37

Supplementary Table S2: Physiochemical properties of LusPLA$_2$s computed by using Expasy's ProtParam tool.

Enzyme	AA	Mol wt (kDa)	pI	Instability Index	Aliphatic Index	GRAVY
LusPLA$_2$I	166	17.94	6.68	34.33	95.72	0.110
LusPLA$_2$II	144	15.72	8.84	17.13	69.79	-0.101

* Instability index lower than 40 classifies the protein as stable.

Supplementary Table S3: Amino acid composition of $sPLA_2$ from flax.

**$LusPLA_2I$**				
%A: 6.0	%C: 7.8	%D: 6.0	%E: 3.0	%F: 1.8
%G: 7.8	%H: 4.8	%I: 3.0	%K: 5.4	%L: 15.1
%M: 2.4	%N: 3.6	%P: 4.2	%Q: 2.4	%R: 3.0
%S: 9.6	%T: 3.6	%V: 6.6	%W: 0.0	%Y: 3.6
**$LusPLA_2II$**				
%A: 6.2	%C: 8.3	%D: 5.6	%E: 2.8	%F: 6.9
%G: 10.4	%H: 2.1	%I: 5.6	%K: 9.7	%L: 7.6
%M: 2.1	%N: 4.9	%P: 2.8	%Q: 2.8	%R: 3.5
%S: 7.6	%T: 4.2	%V: 4.2	%W: 0.7	%Y: 2.1

Supplementary Table S4: Different classes of β-turns in LusPLA$_2$I.

S.No.	Class of β-turns	Position
1	Class I	Thr14-Lys17
		Val30-Leu33
		Pro31-Leu34
		Gly41-Tyr44
		Phe98-Ser101
2	Class II	Cys47-Glu50
3	Class II'	Tyr36-Tyr39
4	Class IV	Ala7-Gln10
		Ser16-Cys19
		Ser25-Cys28
		Phe27-Val30
		Gly37-Cys40
5	Class VIII	Cys28-Pro31
		Pro52-Gly55
		Asn72-Leu75

Supplementary Table S5: Different classes of β-turns in LusPLA$_2$II.

S.no.	Class of β-turns	Position
1	Class I	Aln2-Arg5
		Thr3- His6
2	Class II	Cys39-Glu42
3	Class IV	Ser1-Asn4
		Gly7-Ala10
		Thr15-Val18
		Tyr28-Tyr31
		Gly29-Lys32
		Gly33-Trp36
		Gly60-Gly63
		Val80-Ser83
		Asn82-Lys85
4	Class VIII	Cys16-Gln19

Supplementary Table S6: Details of protein sequences of $sPLA_2s$ from flax.

S.no.	Enzymes	EC. no.	Accession no.	Length (a.a.)	Group	Predicted Localization
1	$LusPLA_2I$	3.1.1.4	KU361324	166	XIB	Extracellular space/ Mitochondria
2	$LusPLA_2II$	3.1.1.4	KU361325	144	XIA	Extracellular space

Supplementary Table S7: List of primers.

**Name**	**Primer sequence**
LusPLA$_2$I F EC	5'-CACCATGGCTGCTTCTTCTGGAATAC-3'
LusPLA$_2$I R EC	5'-AGGCCTGTGGAGGTATCTAC-3'
LusPLA$_2$II F EC	5'-CACCATGAGGTTTAATGGTCTG-3'
LusPLA$_2$II R EC	5'-TAAAAGAGAATTCGCAAAGAATC-3'
